# Understanding the serial mediating effects of career adaptability and career decision-making self-efficacy between parental autonomy support and academic engagement in Chinese secondary vocational students

**DOI:** 10.3389/fpsyg.2022.953550

**Published:** 2022-08-10

**Authors:** Ruyi Jiang, Ruomeng Fan, Yue Zhang, Yunxing Li

**Affiliations:** Research Center for Comprehensive Improvement of High-Quality Education, Institute of Teacher Education, Zhejiang Normal University, Jinhua, China

**Keywords:** parental autonomy support, academic engagement, secondary vocational students, career adaptability, career decision-making self-efficacy

## Abstract

This study investigated new avenues for understanding the association between parental autonomy support and academic engagement among Chinese secondary vocational students based on Self-Determination Theory and Career Construction Theory. We highlighted the mediator role of career adaptability and career decision-making self-efficacy in the relationship between parental autonomy support and academic engagement. Using self-reported data from 1,930 secondary vocational students in a city in Central China, we performed correlation analysis and mediation analysis by using SPSS and Mplus. The results revealed that parental autonomy support was positively associated with students’ academic engagement. Moreover, as an adaptability resource and adapting response, career adaptability and career decision-making self-efficacy played mediating roles between parental autonomy support and academic engagement. These findings offered crucial empirical evidence for understanding the association between parental support and academic engagement among Chinese secondary vocational students. Meanwhile, it also validated the application of Career Construction Theory in a sample of secondary vocational students in China and provided constructive insights for implementing diverse support measures to boost their academic and career development.

## Introduction

Academic engagement refers to the cognitive, behavioral, and emotional states that is continuous, pleasant, and fulfilling developed by the individual toward learning (Schaufeli et al., 2002). It is an essential indicator of students’ academic achievement and can profoundly influence students’ future success (Anderman and Patrick, 2012). Moreover, academic engagement is also associated with lower dropout rates (Archambault et al., 2009). Academic engagement assists students, regardless of backgrounds, in achieving their educational goals, thereby increasing their prospects of future success (Kuh, 2009). According to Peng et al. (2022), academic engagement is a state of being that can be changed and highly influenced by family factors. Parents are a crucial environmental component in the development of individuals (Johnston, 2018), and parental support plays a vital role in facilitating the internalization of students’ learning motivation (Ryan and Deci, 2000).

Many studies have confirmed that parental autonomy support positively correlates with students’ autonomous motivation, sense of competence, and persistence in learning (Assor et al., 2004; Grolnick et al., 2007; Gillet et al., 2012). With sufficient parental support, children are found to be more motivated in their learning, thereby having better academic performance (Grolnick, 2009; Gillet et al., 2012). However, previous studies on academic engagement tended to focus on students in ordinary high schools or universities (Amponsah et al., 2018) and overlooked those in secondary vocational schools (Peng et al., 2022). In China, academic achievement is still a vital index to predict students’ academic quality (Yongsheng and Lejun, 2019). Since a considerable proportion of students enter secondary vocational schools as a result of previous academic failure experiences, the academic engagement of students in these schools has been found to be less than satisfactory in general (Yuejian, 2007). Therefore, they are particularly in need of support and encouragement from parents in order to stay motivated in their academic pursuits (Nini and Meilin, 2013). Thus, it is crucial to comprehend the impact of parental autonomy support on Chinese secondary vocational students’ academic engagement.

Secondary vocational students are vital reserves of primary and intermediate technical talents in the future labor market (Guirong and Jiajia, 2020). In contrast to the traditional learning environment in ordinary high schools, secondary vocational students are trained in both real and simulated work scenarios, which means they have the opportunity to deal with career-related obstacles earlier than their peers (Wang, 2013). To cultivate more advanced-level technical and vocational talents, China has been actively promoting the reform of secondary vocational education. This reform policy emphasizes the same priority of vocational education as general education, therefore opening up various advancement paths for secondary vocational students on the policy level and providing them with more development possibilities (Weichen, 2022). Meanwhile, career adaptability and decision-making self-efficacy are widely acknowledged as crucial psychological resources for coping with future career obstacles and facilitating career advancement in unpredictable circumstances (Savickas and Porfeli, 2012; Duffy et al., 2015; Johnston, 2018). Therefore, career psychological resources are vital in helping secondary vocational students adjust to and benefit from the new environment created by the reform of secondary vocational education.

Previous studies have found a positive correlation between students’ career adaptability and academic engagement (Negru-Subtirica and Pop, 2016). Students are likely to be more motivated to devote efforts to their studies in order to reach higher career goals if they anticipate a better future (Gollwitzer, 1996; Lapan, 2004). Perry also indicated that higher level of career decision-making self-efficacy predicted higher level of school engagement (on behavioral and psychological levels) (Perry, 2008). Nevertheless, in contrast to the positive correlation found between career psychological resources and academic performance, most prior studies concerning Chinese secondary vocational students have only focused on either vocational or academic factors, rather than combining the two for consideration (Zhen, 2014; Xiao and Lijie, 2021). This study is a novel attempt in this regard. We included both vocational and academic factors into the model to examine the internal relationship between parental support and academic engagement of secondary vocational students from the career construction perspective.

## Theoretical framework and research hypothesis

Self-Determination Theory (SDT) pointed out that people tend to engage in work and complete tasks in accordance with their values and interests, but at the same time, people’s motivation and behavior are also affected by the social environment to a certain extent (Ryan and Deci, 2000). When the external environments can satisfy an individual’s sense of competence, ability, and belonging, it helps stimulate his or her innate internalization and integration tendency, thus transforming the external rules and requirements into the value of the individual’s inner identity (Vansteenkiste et al., 2006). In contrast, when a controlling or restrictive social environment suppresses the individual’s inherent motivation and will, it is difficult for him or her to adapt and grow (Ryan and Deci, 2000). Therefore, SDT provides important theoretical support for explaining the influence of the external environment (supportive environment) on individual motivation and behaviors (in terms of academic pursuits and career).

Career adaptation is a crucial skill for secondary vocational students in the vocational transition period, as it enables young adults to manage career-related tasks and transitions and adapt to social changes (Savickas and Porfeli, 2012). According to the Career Construction Theory (CCT), individuals with higher career adaptability have larger capacity for substantial transformation and more psychological resources (Savickas, 1997). In this regard, CCT proposed a Career Construction Model of Adaptation (CCMA) that explained the dynamic development process of an individual’s career adaptability during a series of transitions from school to work (Savickas, 2005, 2013). In the CCMA, the adaptation process includes a sequence of adaptive readiness, adaptability resources, adaptation responses, and adaptation results (Savickas and Porfeli, 2012; Savickas, 2013). Specifically, adaptive readiness increases adaptability resources, adaptability resources shape adaptation responses, and adaptation responses lead to adaptation results. These four dimensions together form an optimal sequence for choosing or entering a particular profession and bridging the transition from one school to another, or from school to work (Savickas et al., 2018).

Secondary vocational students are in the transition stage from school to work (Arum and Shavit, 1995). Especially in the current context of Chinese secondary vocational education reform, their academic and career development have become closely interwoven (Guoqing, 2020). Previous research has found that career adaptation can promote positive academic outcomes, such as academic satisfaction, academic persistence and performance (Duffy et al., 2015; Negru-Subtirica and Pop, 2016; Wilkins-Yel et al., 2018). These findings provide necessary theoretical basis for explaining the relationship between career adaptive psychological resources and academic behaviors.

### The relationship between parental autonomy support and academic engagement

Ryan and Deci (2000) pointed out that a supportive learning environment can stimulate students’ internal motivation and initiative in learning. Supportive environments include close relationships established with family members, teachers, and peers. The family environment is the first important micro-environment for individual growth (Zheng et al., 2021). It has been reported that parents who are able to provide high autonomy support can help children explore and practice their values and interests (Ryan et al., 1995; Clark and Ladd, 2000). For example, students will be more likely to be interested, engaged, and self-assured in their academic endeavors if they believe that their parents have high expectations and recognize their academic accomplishments (Marchant et al., 2001).

In China, secondary vocational students have generally been regarded as a group of students with relatively weak cultural foundations, insufficient learning enthusiasm, and poor self-control ability (Xue and Li, 2021). Negative evaluations from the general public have been found to lead to low academic self-efficacy in secondary vocational students (Yuejian, 2007). A study from Afghan indicated that students who experienced academic stress could still attain higher level of mental wellbeing if they received solid emotional support (Green et al., 2021). Social support may lessen the negative effect of external stressors on individuals (Coleman and Iso-Ahola, 1993). Therefore, for secondary vocational students who have usually suffered more academic setbacks (Yuejian, 2007), support and recognition from their parents would help alleviate the negative impact of the external environment on their academic motivation and help reassure them of their self-worth.

**Hypothesis 1: Based on the aforementioned findings, we propose that parental support is positively associated with the academic engagement of secondary vocational students in China.**

### The independent mediating roles of career adaptability and career decision-making self-efficacy

Career adaptability refers to an individual’s ability to self-regulate when addressing unfamiliar, complex, and uncertain issues in career development tasks, career role transitions, and work trauma (Savickas and Porfeli, 2012). It is considered an essential psychological resource for individuals to successfully transition from school to work (Koen et al., 2012). Career adaptability has been shown to be associated with a host of important outcomes, such as a higher level of academic satisfaction (Wilkins et al., 2014), higher employment quality (Koen et al., 2012), and greater career success (de Guzman and Choi, 2013). Academic engagement is regarded as a consequence of possessing career adaptability resources (Merino-Tejedor et al., 2016; Savickas et al., 2018; Šverko and Babarovic, 2019). Students with greater career adaptability tend to be more proactive in meeting academic demands and rising to challenges, thus increasing the likelihood of academic persistence (Wilkins-Yel et al., 2018). Therefore, we believe that career adaptability for Chinese secondary vocational students will also be positively related to their academic engagement.

In addition, prior research has indicated that career adaptability is positively associated with perceived social support, particularly parental support (Ginevra et al., 2015; Hui et al., 2018). During the initial phases of vocational growth and exploration, parental support is thought to be of significant influence and importance to the development of career adaptability (Guan et al., 2016). The more support parents provide, the better the development of children’s career adaptability (Öztemel and Yıldız-Akyol, 2021). Secondary vocational students, transitioning from youth to adulthood and from school to work, are at the stage of forming work-related values and interests, as well as exploring career options (Wang, 2013; Rodríguez et al., 2016). Therefore, parental support is essential to the development of secondary vocational students’ career adaptability.

**Hypothesis 2: In light of the above analysis, we propose that career adaptability can mediate the relationship between parental autonomy support and academic engagement of secondary vocational students.**

Career decision-making self-efficacy (CDMSE) is the individual’s belief in his or her ability to complete decision-making tasks associated with his or her career successfully (Betz et al., 1996). It is derived from Bandura’s social cognition theory (Locke, 1987) and is an essential factor affecting an individual’s career development (Xing and Rojewski, 2018). High CDMSE individuals are more likely to explore and plan their careers, identify their job interests, and work toward their career objectives (Rogers and Creed, 2011). Similar to the role of career adaptability, CDMSE is generally regarded as an adaptive response, which can affect individuals’ adaptation results, such as academic participation (Savickas, 2005, 2013; Perry, 2008; Savickas et al., 2018). As a result, as a vital career psychosociology resource, CDMSE may encourage secondary vocational students to stay engaged in their studies to accomplish higher professional aspirations.

External circumstances can also impact individuals’ subjective sense of career decision-making self-efficacy (Johnston, 2018). Particularly in collectivist cultures, family influences play a significant role in individual career decisions (Tang et al., 1999). Existing research indicated that parental support can enhance career decision-making self-efficacy (Restubog et al., 2010; Xing and Rojewski, 2018; Li et al., 2022) and mitigate the impact of career-related obstacles (Ong et al., 2006). When secondary vocational students encounter work-related obstacles and challenges, parents may assist them in achieving their desire for autonomy by offering emotional support and verbal encouragement, thereby increasing their willingness in career exploration and confidence in making career-related decisions (Metheny et al., 2008; Garcia et al., 2015).

**Hypothesis 3: On the basis of this analysis, we propose that career decision-making self-efficacy can mediate the relationship between parental autonomy support and academic engagement of secondary vocational students.**

### The chain mediating role of career adaptability and career decision-making self-efficacy

Career construction theory (CCT) has been developed to provide a unifying framework for a better understanding of the relationship between career adaptability and career decision-making self-efficacy (Savickas, 2002). According to the model, career adaptability and career decision-making self-efficacy were considered as adaptability resources and adapting responses, respectively (Savickas and Porfeli, 2012; Savickas, 2013; Hirschi et al., 2015). The CCMA further posited that greater levels of career adaptation were achieved by individuals who were willing (adaptive readiness) and able (adaptability resources) to make successful adaptive responses (Savickas, 2002; Savickas and Porfeli, 2012). Empirical findings from the study conducted by Stead et al. (2021) confirmed the positive relationship between career adaptability and career decision-making self-efficacy. Career adaptability can assist individuals in capitalizing on their strengths, such as self-efficacy, and furthering their career planning, coping skills, and self-regulation behaviors (Johnston, 2018).

**Hypothesis 4: In light of the analysis above, we propose that career adaptability and career decision-making self-efficacy play the role of a chain mediator in the relationship between parental autonomy support and academic engagement of secondary vocational students.**

### The research hypothesis model

**FIGURE 1:**
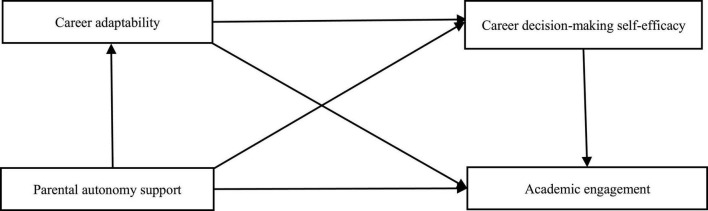
Research hypothesis model.

This study intends to understand the relationship between parental autonomy support and Chinese secondary vocational students’ academic engagement and its internal mechanism by examining parental autonomy support, academic engagement, career adaptability, and career decision-making self-efficacy simultaneously in a model based on self-determination theory and career construction theory. It is expected to offer practical insights in boosting Chinese secondary vocational students’ academic engagement and developing their capacity to deal with career challenges. Additionally, since gender, grade, and family financial status may impact academic engagement and career decision-making self-efficacy (Lam et al., 2012; Xing and Rojewski, 2018), we propose the following hypothesis model (Figure 1) while controlling for these demographic factors.

## Materials and methods

### Participants

This study followed the ethical principles of scientific research. Our study has been approved by the first author’s university and the presidents of the participating schools. Participation in the survey was voluntary and anonymous. Participants were informed in advance that their responses in the questionnaire would be used anonymously and for research purposes only.

In this study, 2,168 Chinese secondary vocational students were selected as subjects from a comprehensive secondary vocational school in a city in Central China using a random sampling method. After eliminating invalid questionnaires and missing subjects, 1,930 valid questionnaires were collected, with a recovery efficiency of 87.45%. Among the respondents of the valid questionnaires, 649 (34.2%) were female students and 1,247 (65.8%) were male students. There were 1,021 (53.9%) respondents from the fourth grade, 809 (42.7%) from the second grade, and 66 (3.5%) from the third grade. The mean age was 15 ± 0.90 years. The demographic information of participants is shown in Table 1.

**TABLE 1:** Demographic characteristics of the sample.

Demographic variables	Category	Frequency (people)	Percentage
Gender	Male	1,255	65%
	Female	675	35%
Grade	First grade	1,039	53.8%
	Second grade	818	42.4%
	Third grade	71	3.7%
	Other	2	0.1%
Family financial status (SES)	Level 1	66	3.4%
	Level 2	71	3.7%
	Level 3	263	13.6%
	Level 4	432	22.4%
	Level 5	739	38.3%
	Level 6	253	13.1%
	Level 7	65	3.4%
	Level 8	25	1.3%
	Level 9	2	0.1%
	Level 10	14	0.7%

Family financial status options from level 1 to level 10 represent family financial status from worst to best.

### Measures

This study used different scales to investigate secondary vocational students’ parental autonomy support, academic engagement, career adaptability, and career decision-making self-efficacy.

#### Parental autonomy support scale

We used a Chinese version of the “Parental Autonomy Support” scale (Qin et al., 2013). It contained 12 items, such as “My parents let me make my own choices whenever possible” and “My parents encourage me to express my ideas when making decisions about me.” Qin et al. (2013) have verified the scale with Chinese high school students as subjects and found that the scale had good reliability and validity. The measurement items used a 5-point Likert scale. Students responded to each item by indicating how true it was describing their parents (1 = “not at all true” to 5 = “very true”) and the total score of the 12 items were taken, with higher numbers indicating greater support for autonomy. The Cronbach’s α of the scale used in this study was 0.95, indicating good reliability.

#### Academic engagement scale

The Academic Engagement scale used in this study was translated and revised from the Student Engagement Questionnaire prepared by Lam et al. (2012). The scale consisted of three dimensions and 16 items. These dimensions covered behavioral engagement, emotional engagement, and cognitive engagement. Behavioral engagement included five questions, such as “I study hard in class”; emotional engagement included five items, such as “Class is fun”; cognitive engagement consisted of six questions, such as “In the process of learning, I always relate new knowledge to my own experience.” The measurement items used a 5-point Likert scale with 1 for “strongly disagree” and 5 for “strongly agree,” and a higher overall score indicated better academic engagement. Zheng et al. (2021) have tested the scale on Chinese students and found that the scale had good reliability and validity. The Cronbach’s α of the scale used in this study was 0.98, indicating good reliability.

#### Career adaptability scale

The Career Adaptability scale used in this study was simplified from the career adaptability scale developed by Savickas and Porfeli (2012). The revised scale had 12 questions, including four dimensions: concern, control, confidence, and curiosity. Career concern included three questions, such as “Thinking about what my future will be like”; career control consisted of three items, such as “Making decisions by myself”; career confidence included three questions, such as “Learning new skills”; career curiosity included three questions, such as “Observe different ways of doing things.” The measurement items used a 5-point Likert scale (1 = “not strong” to 5 = “extremely strong”) and a higher overall score indicated better career adaptability. The Cronbach’s α of the scale used in this study was 0.97, indicating good reliability.

#### Career decision-making self-efficacy scale

This study used the Chinese version of the Career Decision-Making Self-efficacy scale to assess self-efficacy in career decision-making (Betz et al., 1996). The scale contained 25 items measuring respondents’ confidence in performing tasks related to five career choice competencies: self-appraisal, information gathering, goal selection, planning, and problem-solving. Examples of the items were “Persist in pursuing your professional (career) goals even when you encounter various difficulties” and “Develop a study plan to improve grades in subjects relevant to your future major (career).” The scale asked respondents to rate their confidence level using a 5-point Likert scale (1 = “not confident at all” to 5 = “completely confident”). This scale has been used widely and had good reliability and validity (Creed et al., 2009). Previous research has tested and validated the Chinese version of this scale (Zhou et al., 2016). The Cronbach’s α of the scale used in this study was 0.98, indicating good reliability.

### Procedure and data analysis

We had obtained permission from participants and their parents before they completed the questionnaire during the class. The completion process was supervised by a research assistant trained in standardized questionnaire administration procedures. SPSS 26.0 and Mplus version 8.3 were used for descriptive analysis, correlation analysis, and mediation analysis. Since gender, grade, and family socioeconomic status could influence academic engagement, parental autonomy support, and career decision-making self-efficacy (Lam et al., 2012; Xing and Rojewski, 2018), these factors were included as control variables in the analysis. The Human Experimentation Ethics Committee of Zhejiang Normal University has approved this project.

## Results

### Descriptive statistics

Table 2 presents means, standard deviations, and correlations for all study variables. The Pearson correlation among parental autonomy support, career adaptability, career decision-making self-efficacy, and academic engagement revealed significant positive associations with one another.

**TABLE 2:** Descriptive statistics of each variable and the results of their correlation analysis.

Variables	*M* ± *SD*	1	2	3	4	5	6	7
1. Parental autonomy support	41.18 ± 8.74	−						
2. Career adaptability	35.23 ± 9.92	0.30***	−					
3. Career decision-making self-efficacy	77.58 ± 15.27	0.41***	0.57***	−				
4. Academic engagement	50.68 ± 10.24	0.36***	0.53***	0.75***	−			
5. Gender	1.35 ± 0.48	0.02	−0.10***	−0.01	0.01	−		
6. Grade	1.50 ± 0.57	−0.01	0.02	−0.03	0.002	−0.11***	−	
7. SES	4.53 ± 1.42	0.08**	0.03	0.03	0.07**	−0.03	−0.07**	−

*n* = 1930, *p < 0.05, **p < 0.01, ***p < 0.001.

### The mediation model

**FIGURE 2:**
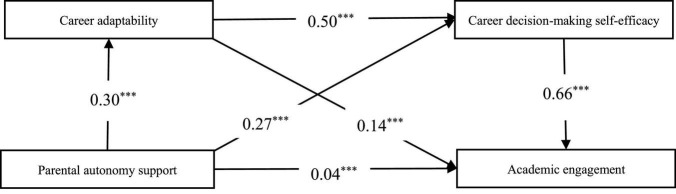
Chain intermediary model. The *** indicates that the mediation effect path coefficient is significant at the 0.001 level.

The standardized regression coefficients for all paths of the mediation model are shown in Figure 2. The goodness of the model fit was satisfied, as shown by the following model fit indicators: χ^2^/*df* = 2.134, RMSEA = 0.024, SRMR = 0.021, CFI = 0.996, TLI = 0.991. The result indicated that parental autonomy support was positively associated with career adaptability (β = 0.30, *p* < 0.001) and academic engagement (β = 0.04, *p* < 0.001), and career adaptability was positively associated with academic engagement (β = 0.14, *p* < 0.001). Career adaptability partially mediated the association between parental autonomy support and academic engagement (indirect effect = 0.04, 95% CI = [0.03, 0.06], accounting for 11.43% of the total effect). Besides, parental autonomy support was also positively associated with career decision-making self-efficacy (β = 0.27, *p* < 0.001), and career decision-making self-efficacy was significantly and strongly associated with academic engagement (β = 0.66, *p* < 0.001). Career decision-making self-efficacy partially mediated the association between parental autonomy support and academic engagement (indirect effect = 0.17, 95% CI = [0.14, 0.21], accounting for 48.57% of the total effect). In the same vein, career adaptability was also positively associated with career decision-making self-efficacy (β = 0.50, *p* < 0.001). Results denoted that career adaptability and career decision-making self-efficacy played the role of a serial mediator in the relationship between parental autonomy support and academic engagement (indirect effect = 0.10, 95% CI = [0.08, 0.12], accounting for 28.57% of the total effect).

### The intermediate effect test

A bias-corrected percentile Bootstrap (repeated sampling 2000 times) was applied, and the result of mediating analysis is shown in Table 3.

**TABLE 3:** Results of intermediate effect test.

Mediation paths	Standardized indirect effect estimates	Effectiveness ratio	Confidence interval	
			LL	UL
Direct effect	0.04***	11.43%	0.01	0.08
Total indirect effect	0.31***	88.57%	0.27	0.36
Ind1: Parental autonomy support → Career adaptability → Academic engagement	0.04***	11.43%	0.03	0.06
Ind2: Parental autonomy support → Career decision-making self-efficacy → Academic engagement	0.17***	48.57%	0.14	0.21
Ind3: Parental autonomy support → Career adaptability → Career decision-making self-efficacy → Academic engagement	0.10***	28.57%	0.08	0.12

*n* = 1930, *p < 0.05, **p < 0.01, ***p < 0.001; LL, lower 2.5% confidence interval; UL, upper 2.5% confidence interval.

## Discussion

Based on the self-determination theory (SDT) (Ryan and Deci, 2000) and career construction theory (CCT) (Savickas, 2005), we investigated new avenues for understanding the association between parental autonomy support and academic engagement among Chinese secondary vocational students. Specifically, we examined the relations among parental autonomy support, career adaptability, career decision-making self-efficacy, and academic engagement. As predicted, parental autonomy support was positively associated with academic engagement. The relations between parental autonomy support and academic engagement were mediated by career adaptability and career decision-making self-efficacy. These findings are consistent with our expectations and also with the predictions made by the SDT and CCT. In the following paragraphs, we briefly discussed the contributions of these significant findings to previous research and theories.

Our result verified that parental autonomy support was positively correlated with academic engagement in a sample of Chinese secondary vocational students (**H1**). Consistent with existing literature linking parental support to academic engagement (Peng et al., 2022), this result confirms that the external environment played an important role in the change of individuals’ intrinsic motivation and behavior as elaborated by SDT (Ryan and Deci, 2000). This suggests that for secondary vocational students who tend to experience higher academic dissatisfaction, parental support and recognition effectively may mitigate the external environment’s negative influences on academic motivation and promote their academic engagement (Wenzhu et al., 2016).

Our study also found that career adaptability and career decision-making self-efficacy partially mediated the correlation between parental autonomy support and academic engagement (**H2** and **H3**). It supported the SDT by proving that an environment providing autonomy support could stimulate the individual’s autonomous explorative behaviors (Ryan and Deci, 2000; Assor et al., 2004). This may be due to the distinctive attitude toward the family in Chinese culture, in which “social norms value affiliation, interdependence, and respect for elders, [therefore] individuals rely strongly on family for support throughout their lifetime” (Xing and Rojewski, 2018, p. 48). Chinese people could be more inclined to consider family expectations and obligations when choosing career (Hannum et al., 2011). Thus, parents play a crucial role in encouraging adolescents to explore their career interests and goals. Additionally, career adaptability and career decision-making self-efficacy have been validated as specific performance measuring individual adaptability resources and adapting responses that could lead to positive adaptation results (in terms of academic satisfaction and academic engagement) (Savickas, 2005, 2013; Savickas and Porfeli, 2012; Rudolph et al., 2017). This provided support for the CCMA model in explaining the career development process of secondary vocational students in China.

In addition to the above findings, we also found that career adaptability and career decision-making self-efficacy played the role of a serial mediator between parental autonomy support and academic engagement (**H4**). The mediating effect of this sequence suggested that parental autonomy support enabled children to have more adaptability resources (career adaptability), which led to better adapting responses (career decision-making self-efficacy) and ultimately affected their adaptation results (academic engagement). For the first time, this series of mediating chains verify the role of the CCMA in the relationship between parental autonomy support and academic engagement and reveals the internal mechanism. This provides crucial empirical support for the development of CCT (Savickas, 2005, 2013).

Among them, the most exciting finding of this study was that the mediating effect of career decision-making self-efficacy and the serial mediating effect accounted for the most significant proportion of the total effect size, indicating that parental autonomy support had the most proactive correlation with the academic engagement of secondary vocational students through the mediating effect of career adaptability and career decision-making self-efficacy. This was possible because vocational education valued the cultivation of students’ vocational skills (Wang, 2013), and the academic self-efficacy of secondary vocational students was in general relatively low due to previous failed academic experiences (Wenzhu et al., 2016). Therefore, they might need a higher level of career confidence to maintain their enthusiasm and commitment to their study. This is consistent with the result of a longitudinal study conducted by Negru and Pop, which found an interaction between career adaptability and academic performance. Career adaptability reflected individuals’ expectations for future careers. Adolescents with strong future orientation were usually full of confidence in their career development and more focused and engaged in their studies with the aim of achieving better academic performance (Negru-Subtirica and Pop, 2016). This finding also provided positive inspiration for adopting various support strategies to promote secondary vocational students’ academic and career development.

## Implications and limitations

### Practice implications

Our result showed that parental autonomy support was positively correlated with the academic engagement of secondary vocational students. Parental support, affirmation, encouragement, and admiration are vital emotional resources in a person’s maturation process (Wenzhu et al., 2016). Parents with high autonomy support would respect their children’s wishes, allow them to explore by themselves, and help to mobilize their enthusiasm for learning (Nini and Meilin, 2013). Secondary vocational students are at a critical juncture in developing their sense of self (Rodríguez et al., 2016). Consequently, parents of secondary vocational students should provide more support and trust to their children in order to meet their independent development needs. This helps to enhance children’s initiative, cultivate their ability to choose, and stimulate the development of intrinsic motivation (Grolnick et al., 2007). Especially in the context of secondary vocational education reform providing more opportunities for secondary vocational students’ academic development, parents should respect their children’s choice of future development, believe in their ability, and provide possible academic support, which can help stimulate the development of intrinsic motivations in their academic pursue.

Career adaptability and self-efficacy were widely considered as essential resources for successfully navigating career development and career decision processes (Savickas and Porfeli, 2012; Duffy et al., 2015; Johnston, 2018). Students with more career-adaptive psychological resources would be happier to engage in their education to reach higher career goals because they anticipated a better future (Gollwitzer, 1996; Lapan, 2004). Therefore, in addition to directly influencing the academic engagement of secondary vocational students, parents should also actively pay attention to their children’s career development in order to indirectly strengthen their children’s academic motivation. Secondary vocational students are in a transitional stage of forming work values and interests and exploring career options (Wang, 2013; Rodríguez et al., 2016). Under the background of the reform of secondary vocational education and the continuous improvement of the vocational education system, parents should have higher educational expectations for secondary vocational students, assist their children in making future career plans based on their children’s wishes, and encourage them to strive for a higher educational platform. In addition, parents should validate their children’s career-related abilities, since this may encourage children to be more optimistic and self-assured regarding their future career development.

Given the significance of parental support in the growth of secondary vocational students, secondary vocational education institutions should emphasize the benefits of parental support. Since future-oriented teenagers fare better in terms of career and academic achievement (Negru-Subtirica and Pop, 2016), vocational high schools may seek parental support and cooperation to assist students in developing career plans, making career decisions, and adapting to the change of roles during the school-to-work transition (Xing and Rojewski, 2018). This will assist secondary vocational students in identifying their future career development direction and taking practical steps to accomplish these objectives. Additionally, career service center in schools may organize meetings with parents to inform them of their children’s recent progress, explain the role of parents in students’ career development, and encourage parents to trust their children’s development potentials and allow them more autonomy to explore future career possibilities. Schools that actively strive for the cooperation and support of parents will help secondary vocational students to clarify their future career development goals and stimulate their internal development motivation.

### Limitations and future research

The hypothesis model was tested using a cross-sectional design. Although the structural equation model method has been used to reveal the relationship between parental autonomy support and academic engagement of secondary vocational students, the cross-sectional characteristics of the study means that causal relationships between variables may not be completely reliable. Future research may focus on a longitudinal design. Also, the data in this study were all gathered using self-reported measures, which may have inevitably introduced subject-related biases. The follow-up study will adopt different data collection methods to examine the relationship between the variables, such as evaluating the level of parental autonomy support and academic engagement of secondary vocational students from the parents’ perspective. Finally, the samples of the current study were only collected from one city in China. Future studies can gather data from more different areas to validate the serial mediating effects of career adaptability and career decision-making self-efficacy in the relationship between parental autonomy support and academic engagement among Chinese secondary vocational students to improve the generalizability of study outcomes.

## Data availability statement

The raw data supporting the conclusions of this article will be made available by the authors, without undue reservation.

## Ethics statement

The studies involving human participants were reviewed and approved by Research Ethics Committee of Zhejiang Normal University. Written informed consent to participate in this study was provided by the participants’ legal guardian/next of kin.

## Author contributions

RJ performed the experiments, analyzed the data, and drafted the manuscript. All authors conceived and designed the experiments, revised the manuscript, contributed to the article, and approved the submitted version.
